# The vitamin E analog, alpha-tocopheryloxyacetic acid enhances the anti-tumor activity of trastuzumab against HER2/neu-expressing breast cancer

**DOI:** 10.1186/1471-2407-11-471

**Published:** 2011-11-02

**Authors:** Tobias Hahn, Deborah J Bradley-Dunlop, Laurence H Hurley, Daniel Von-Hoff, Stephen Gately, Disis L Mary, Hailing Lu, Manuel L Penichet, David G Besselsen, Brook B Cole, Tanisha Meeuwsen, Edwin Walker, Emmanuel T Akporiaye

**Affiliations:** 1 Earle A. Chiles Research Institute, Robert W. Franz Cancer Research Center, Providence Portland Medical Center, Portland, OR, 97213, USA; 2 Department of Immunobiology, The University of Arizona, Tucson, AZ, 85724, USA; 3 Department of Pharmacy, The University of Arizona, Tucson, AZ, 85724, USA; 4 The Arizona Cancer Center, The University of Arizona, Tucson, AZ, 85724, USA; 5 Translational Genomics Research Institute, Phoenix, AZ, 857004, USA; 6 School of Medicine, The University of Washington, Seattle, WA, 98195, USA; 7 Division of Surgical Oncology, Department of Surgery, University of California, Los Angeles, CA, 90095, USA; 8 Department of Microbiology, Immunology, and Molecular Genetics, University of California, Los Angeles, CA, 90095, USA; 9 The Molecular Biology Institute, University of California, Los Angeles, CA, 90095, USA; 10 Jonsson Comprehensive Cancer Center, University of California, Los Angeles, CA, 90095, USA; 11 Department of Veterinary Science and Microbiology, The University of Arizona, Tucson, AZ, 85721, USA

## Abstract

**Background:**

*HER2/neu *is an oncogene that facilitates neoplastic transformation due to its ability to transduce growth signals in a ligand-independent manner, is over-expressed in 20-30% of human breast cancers correlating with aggressive disease and has been successfully targeted with trastuzumab (Herceptin^®^). Because trastuzumab alone achieves only a 15-30% response rate, it is now commonly combined with conventional chemotherapeutic drugs. While the combination of trastuzumab plus chemotherapy has greatly improved response rates and increased survival, these conventional chemotherapy drugs are frequently associated with gastrointestinal and cardiac toxicity, bone marrow and immune suppression. These drawbacks necessitate the development of new, less toxic drugs that can be combined with trastuzumab. Recently, we reported that orally administered alpha-tocopheryloxyacetic acid (α-TEA), a novel ether derivative of alpha-tocopherol, dramatically suppressed primary tumor growth and reduced the incidence of lung metastases both in a transplanted and a spontaneous mouse model of breast cancer without discernable toxicity.

**Methods:**

In this study we examined the effect of α-TEA plus HER2/*neu*-specific antibody treatment on HER2/neu-expressing breast cancer cells *in vitro *and in a HER2/*neu *positive human xenograft tumor model *in vivo*.

**Results:**

We show *in vitro *that α-TEA plus anti-HER2/*neu *antibody has an increased cytotoxic effect against murine mammary tumor cells and human breast cancer cells and that the anti-tumor effect of α-TEA is independent of HER2/*neu *status. More importantly, in a human breast cancer xenograft model, the combination of α-TEA plus trastuzumab resulted in faster tumor regression and more tumor-free animals than trastuzumab alone.

**Conclusion:**

Due to the cancer cell selectivity of α-TEA, and because α-TEA kills both HER2/*neu *positive and HER2/*neu *negative breast cancer cells, it has the potential to be effective and less toxic than existing chemotherapeutic drugs when used in combination with HER2/*neu *antibody.

## Background

Alpha-tocopheryloxyacetic acid (α-TEA) is an ether derivative of naturally occurring vitamin E (alpha-tocopherol). Unlike vitamin E, which lacks *in vivo *anti-tumor activity and fails to prevent cancer in humans [1,2], α-TEA is directly cytotoxic to tumor cells [3-7] via a mechanism that includes mitochondrial depolarization and generation of reactive oxygen species leading to apoptotic cell death [8-10] as has been reported for alpha-tocopheryl succinate (α-TOS) [11]. Unlike alpha-tocopheryl succinate (α-TOS), which is susceptible to conversion to the apoptosis-inert tocopherol and succinic acid by intestinal esterases, α-TEA is stable and induces apoptosis of a variety of mouse and human cancer cell lines while sparing normal cells [3,4,6,7]. More importantly, we reported recently that oral α-TEA significantly inhibited the growth of transplanted murine breast cancer (4T1) and dramatically reduced the incidence of lung metastases [7] and was able to suppress growth in a clinically relevant spontaneous model of breast cancer (MMTV-PyMT) without overt toxicity [6].

*HER2/neu *is a proto-oncogene that encodes a 185-kDA tyrosine kinase receptor and is related to members of the epidermal growth factor receptor family [12]. HER2/*neu *promotes neoplastic transformation by virtue of its ability to transduce growth signals in a ligand-independent manner [13,14]. The HER2/*neu *protein is over-expressed in 20-30% of invasive human breast cancers [15,16], is associated with aggressive disease [15,17] and has been successfully targeted in HER2/*neu*^+^, hormone receptor positive or negative, breast cancer patients with trastuzumab (Herceptin^®^) [18-20], which is a humanized monoclonal antibody directed against the extracellular domain of the HER2/*neu *protein. When used as a single agent, trastuzumab is beneficial only in 15-30% of HER2/*neu*^+ ^breast cancer patients that express very high levels of HER2/*neu *protein but efficacy can be enhanced when combined with chemotherapeutic drugs [18,21]. Although trastuzumab is widely used for the treatment of HER2/*neu *over-expressing breast cancers, its mechanism of action is still only partially understood. There is evidence that trastuzumab inhibits proliferation and survival of breast cancer cells by mechanisms that include stimulation of antibody-dependent cell-mediated cytotoxicity (ADCC) [22,23], inhibition of angiogenesis [24,25], and enhancement of endocytic degradation of HER2/*neu*, although the latter finding remains controversial [23].

Given the different mechanisms by which α-TEA and trastuzumab mediate tumor cell death [3,26-29], we hypothesized that combining α-TEA with HER2/*neu*-specific antibody will result in enhanced anti-tumor activity against HER2/*neu*-expressing breast cancer. In this report we evaluated the anti-tumor activity of concurrent α-TEA and anti-HER2/*neu *antibody treatment against HER2/*neu*-expressing murine mammary and human breast cancer cells *in vitro *and on established HER2/*neu*^+ ^human breast cancer in a murine xenograft tumor model. We report that α-TEA induces cell death of several mouse mammary and human breast cancer cell lines irrespective of HER2/*neu *status. More importantly, when combined with anti-HER2/*neu *antibody, α-TEA improves the efficacy of trastuzumab therapy resulting in complete regression of established HER2/*neu*^+ ^human breast cancer xenografts. These results suggest that α-TEA is a viable less toxic agent which can be used in combination with trastuzumab for the treatment of HER2/*neu*^+ ^breast cancer.

## Methods

### Reagents

#### α-Tocopheryloxyacetic acid

Alpha-TEA [(2,5,7,8-tetramethyl-(2R-(4R,8R,12-trimethyltridecyl) chroman-6-yloxy) acetic acid)] was synthesized at The Arizona Cancer Center Synthetic Shared Resource at The University of Arizona (Tucson, AZ) using modified previously described methods [3,30,31]. Purity and identity was confirmed by high-performance liquid chromatography and nuclear magnetic resonance analysis. To make α-TEA soluble in aqueous culture media for *in vitro *assays, α-TEA was vesiculated (Vα-TEA) by sonication of an α-TEA thin film in the presence of PBS as previously described [7].

#### Antibodies

The anti-rat neu antibody (7.16.4) was generated as described previously [32]. The non-specific mouse IgG_2a _antibody was purchased from Chemicon (cat#: PP102, Chemicon Int. Temecula, CA). Trastuzumab (Herceptin^®^) and rituximab (Rituxan^®^) were purchased from Genentech (San Francisco, CA).

### Tumor cells and cell culture

Rat HER2/*neu *positive (MMC) and HER2/*neu *negative (ANV) mouse mammary tumor cell lines [33] and human HER2/*neu *positive (MDA-MB-453) and HER2/*neu *negative (MDA-MB-231) breast cancer cell lines were used for these studies. The human cell lines were purchased from American Type Culture Collection (ATCC, Manassas, VA). The MMC and ANV tumor cell lines were maintained in RPMI-1640 with glucose and L-glutamine (Lonza, Walkersville, MD) containing 100 U/mL penicillin, 100 mg/mL streptomycin (Hyclone Laboratories, Logan, UT), 0.025 mg/mL Amphotericin B (Hyclone), 0.1 mM non-essential amino acids (Lonza), 1 mM sodium pyruvate (Lonza), 2 mM L-glutamine (Lonza) and 10% fetal bovine serum (FBS) (Invitrogen, Carlsbad, CA). The MDA-MB-231 and MDA-MB-453 tumor cell lines were maintained in RPMI-1640 containing 50 μg/mL Gentamicin (Lonza), 0.1 mM non-essential amino acids (Lonza), 1 mM sodium pyruvate (Lonza), 2 mM L-glutamine (Lonza) and 10% FBS (Invitrogen, Carlsbad, CA). All cells were maintained in log phase in a humidified chamber at 37°C with 7% CO_2_.

### *In vitro *studies

To determine the cytotoxic effect of α-TEA, tumor cells were plated in three to six replicates in 96-well plates and allowed to adhere overnight. The cells were then treated with media alone or increasing doses of Vα-TEA for 24 hours (MMC, ANV), 48 hours (MDA-MB-231) or 72 hours (MDA-MB-453). Cell survival was determined using the Thiazyl Blue Tetrazolium Bromide (MTT) assay [34]. Briefly, MTT (Sigma, St. Louis MO) in serum-free media was added to achieve a concentration of 5 mg/mL. After a 2 h incubation (37°C, 7% CO_2_), the plate was centrifuged (500 xg, 5 min), the supernatant removed and the dark-blue metabolite was dissolved in DMSO. Cell killing was then determined by measuring absorption at 560 nm.

In order to determine the combined effect of α-TEA plus anti-HER2/*neu *antibody on cell survival, tumor cells were plated in 6 replicates in 96-well plates and allowed to adhere overnight. The cells were then simultaneously treated with pre-determined sub-optimal doses of α-TEA (20 μM for MMC and ANV, and 10 μM for MDA-MB-453 and MDA-MB-231) and HER2/*neu*-specific antibody (20 μg/mL of the 7.16.4 anti-rat HER2/*neu *antibody for MMC and ANV; 10 μg/mL trastuzumab for MDA-MB-453; 20 μg/mL trastuzumab for MDA-MB-231). The respective isotype antibodies were mouse IgG_2a _antibody (MMC, ANV) and rituximab (MDA-MB-453, MDA-MB-231). After 5 days, surviving cells were assessed by MTT assay. To detect apoptosis, MDA-MB-453 cells were plated in 6-well plates overnight and then treated with 10 μM Vα-TEA and 10 μg/mL trastuzumab (or isotype antibody) for 72 hours. Cells were collected using 10 mM EDTA and stained using the PE-Annexin-V/7AAD apoptosis detection kit (BD Pharmingen, San Diego, CA) according to the manufacturer's instructions and analyzed by flow-cytometry using a LSR-II flow cytometer (BD Bioscience) with data acquisition using DIVA software (BD Bioscience). Cells positively staining with PE-Annexin-V were considered to be apoptotic.

### Western Immunoblotting

MDA-MB-453 tumor cells were plated in 6-well culture dishes overnight and then treated with 10 μM Vα-TEA or 40 μg/mL trastuzumab (or isotype antibody) for 24 h. Cell lysates were prepared using Complete Lysis-M Buffer (Roche Applied Sciences) containing protease inhibitors and phosphatase inhibitors (both Roche Applied Sciences). The lysates were clarified (14,000 xg, 15 min, 4°C) and protein content was determined by BCA (Thermo Scientific, Rockford, IL). Equal amounts of protein (10 μg) were separated on pre-cast TGX-Criterion Any-kd SDS gels (BIO-RAD, Hercules, CA), transferred to Amersham Hybond-ECL nitrocellulose membrane (GE Healthcare). Membranes were blocked with Odyssey Blocking Buffer (LI-COR Biosciences, Lincoln, NE). Primary antibodies used were: anti-phosphorylated-(Ser473)-AKT (1:1000, Cell Signaling Technology, Denvers, MA); anti-AKT (1:1000, Cell Signaling); anti-GAPDH (1:3000, Millipore, Temecula, CA). Primary antibodies were diluted in phosphate-buffered saline (PBS) containing 5% bovine serum albumin (BSA) and 0.1% Tween-20 (PBS/T-5%-BSA). After washing in PBS containing 0.1% Tween-20 (PBS/T), the membranes were exposed to anti-rabbit-HRP-conjugated (Cell Signaling) or anti-mouse-HRP-conjugated (eBioscience, San Diego, CA) secondary antibodies (both 1:10,000). Bands were visualized using SuperSignal WestPico chemiluminescence substrate (Thermo Scientific). Films were scanned and band intensities were quantified using ImageJ software [35].

### Flow cytometry

To determine HER2/*neu *expression in MMC and ANV cells, tumor cells were incubated with the anti-rat HER2/*neu *antibody (7.16.4) and then stained with a FITC-conjugated goat-anti mouse IgG antibody (Caltag, Burlingame, CA). MDA-MB-453 and MDA-MB-231 cells were stained with a FITC-conjugated anti-human erbB2/HER2 antibody (clone 24D2, Biolegend, San Diego, CA). FITC-conjugated isotype antibodies were included as controls. Cells were interrogated using a FACScan flow cytometer (BD Biosciences, San Jose, CA) with data acquisition using CellQuest (BD Biosciences) or a LSR-II flow cytometer (BD Bioscience) with data acquisition using DIVA software (BD Bioscience). Data were analyzed using FlowJo v8.8.4 (Tree Star Inc., Ashland, OR). To determine HER2/*neu *expression *in vivo*, tumors were resected and minced using a scalpel in HBSS (Lonza) containing 5 mM EDTA. The tumor cells were then pushed in succession through metal, 70 μm and 40 μm nylon sieves (BD Biosciences Discovery Labware, Two Oaks, CA). The resulting tumor cell suspension was assessed for cell viability using the LIVE/DEAD Fixable Violet stain (Invitrogen, Carlsbad, CA) and used for flow cytometric analysis of HER2/*neu *expression.

### Animal studies

Six- to eight-week-old female severe combined immunodeficiency (SCID; strain CB17SC) mice were bred at the University of Arizona Cancer Center Experimental Mouse Shared Services (EMSS) or purchased from Taconic Farms Inc. (Hudson, NY). Mice were housed in micro-isolator cages at the animal facilities of either the University of Arizona (Tucson, AZ) or the Earle A. Chiles Research Institute (Providence Portland Medical Center, Portland, OR) in accordance with the Principles of Animal Care (NIH publication No. 85-23). All studies were reviewed and approved by the institutional animal care and use committee (IACUC) of The University of Arizona or the Earle A. Chiles Research Institute.

For the human xenograft studies, 1 × 10^7 ^viable MDA-MB-453 breast cancer cells were injected s.c. into the right mammary fat pad of SCID mice. After tumor establishment (day 15 post-tumor cell injection), the mice remained on standard (nutrient-matched) chow or were transferred to chow containing 1 g/kg α-TEA (0.1% diet, Harlan Teklad) resulting in an intake of ~2 mg of α-TEA per day per mouse (equivalent to ~100 mg α-TEA per kg body weight). The mice were maintained on the α-TEA diet until day 63 post-tumor implantation and were then transferred to standard chow. Trastuzumab (40 μg per injection) or isotype control (rituximab) were administered by intraperitoneal (i.p.) injection on days 15, 17, 19, 22, 24, 26, 29, 31, 33, 36, 38, 40 post-tumor transplantation. Tumor growth was monitored by measuring the tumor length (L) and width (W) using calipers and calculating the tumor volume as: V = (L × W × W/2).

### Histology and determination of *in vivo *cell proliferation and apoptosis

Tumors were resected on day 30 post-tumor injection and tissues were fixed in 10% buffered formalin for 24 h, paraffin-embedded, and 3 μm sections were stained with hematoxylin and eosin (H&E) and evaluated by a veterinary pathologist. Cell proliferation was determined on de-paraffinized 3 μm sections using antibodies specific for the nuclear antigen Ki-67 (Novocastra Laboratories, Newcastle upon Tyne, UK, cat#: NCL-ki67p). Deparaffinization, antigen retrieval and primary antibody staining, detection and amplification of the primary antibody, and hematoxylin counterstaining was performed on a Discovery XT Automated Immunostainer (Ventana Medical Systems, Tucson, AZ) using Ventana Medical Systems validated reagents. Ki-67 was detected using an anti-rabbit biotinylated secondary antibody and by using biotinylated-streptavidin-HRP and a DAB (diaminobenzidine) system (DAPMap). Apoptosis was determined on deparaffinized 5 μm sections by terminal deoxynucleotidyl transferase-mediated dUTP nick end labeling (TUNEL). The TUNEL assay was performed using the ApopTag Fluorescein In Situ Apoptosis Detection Kit (Millipore, Temecula, CA) according to the manufacturer's protocol. Sections were counterstained with propidium iodide in Antifade (Millipore). Sections were evaluated using a Nikon Eclipse TE2000-S inverted microscope (Nikon Instruments Inc., Melville, NY) with epifluorescence capabilities using the B-2E/C filter set (excitation: 465-495 nm band pass; dichromatic mirror: 505 nm long pass; emission: 515-555 nm band pass) and the Y-2E/C filter set (excitation: 540-580 nm band pass; dichromatic mirror: 595 nm long pass; emission 600-660 nm band pass). Pictures were acquired using a DS-Fi1 color camera (Nikon Instruments Inc.) and the Nikon Imaging System Basic Research (NIS-Br, version 3.2) software (Nikon Instruments Inc.). Ki-67 or TUNEL positive cells were counted in 5 microscopic fields (40× magnification) per section from three mice per treatment group using the NIS-Br software.

### Statistical analysis

Statistical significance of differences among data sets of treatment groups was assessed either by Student's *t*-test, where applicable, or by one-way analysis of variances (ANOVA), including Tukey-Kramer post tests for multiple comparisons. Analyses were performed using Prism software (GraphPad, San Diego, CA). Probability values (P) of < 0.05 were considered indicative of significant differences between data sets. For the xenograft studies, mean tumor size across the study time points for the trastuzumab and trastuzumab+α-TEA groups was modeled using a nonlinear mixed model. The mean size was represented by a 4-parameter logistic regression model, with separate slope and lower asymptote parameters for the different treatment groups. Due to the intra-animal correlation across time, a random lower asymptote was inserted into the model to allow the remission curves of different animals to vary. Model estimation was performed using PROC NLMIXED in SAS v. 9.2 (SAS, Cary, NC). The percentage of tumor-free animals at day 63 was compared between groups using Fisher's exact test. Hochberg's method was used to adjust the significance level for multiple comparisons. The absence of tumor across the study time points for the trastuzumab and trastuzumab+α-TEA groups was modeled using generalized estimating equations. The model with a logit link had different linear slopes and a common quadratic slope across days. Model parameters were assessed for significance using empirical standard error estimates. The intra-subject covariance matrix was autoregressive with order 1.

## Results

### α-TEA induces cell death of mouse mammary and human breast cancer cells independent of HER2/*neu *expression

**Figure 1:**
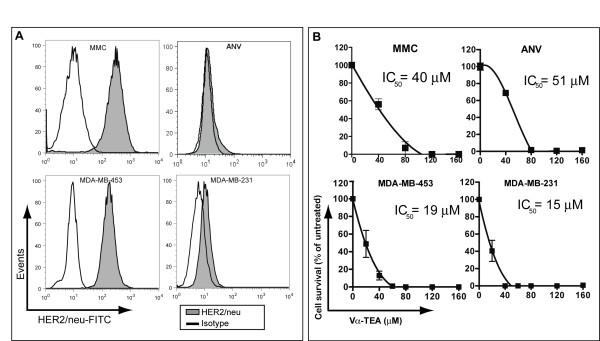
**Murine and human tumor cells are susceptible to α-TEA irrespective of HER2/*neu *status**. (A) HER2/*neu *expression was determined by flow cytometry. The murine MMC and ANV tumor cells were stained with a rat-HER2/*neu*-specific antibody (7.16.4) and then with a FITC-conjugated anti-mouse-IgG antibody. The human MDA-MB-453 and MDA-MB-231 tumor cells were stained using a FITC-conjugated human-HER2/*neu*-specific antibody. Cells were gated on light scatter. (B) Tumoricidal effect of Vα-TEA. Tumor cells were allowed to adhere overnight and were then treated with media alone or increasing doses of Vα-TEA. Cell survival was determined by MTT assay after 24 h (MMC, ANV), 48 h (MDA-MB-231) or 72 h (MDA-MB-453). Results are expressed as mean percentage ± SD of untreated cells (media alone). Non-linear regression analysis was performed to determine half-maximal inhibitory concentrations (IC_50_).

In initial studies, we evaluated the expression level of surface HER2/*neu *in several mouse and human breast cancer cell lines as well as their susceptibility to α-TEA treatment. HER2/*neu *expression was confirmed by flow cytometry in the MMC, and MDA-MB-453 cell lines (Figure 1A). In contrast, the ANV and MDA-MB-231 cell lines expressed minimal HER2/*neu *(Figure 1A). To ascertain the susceptibility of tumor cells to α-TEA, they were exposed to increasing doses of α-TEA and cell survival was determined. The data (Figure 1B) show that irrespective of HER2/*neu *status, both mouse mammary and human breast cancer cells are susceptible to α-TEA in a dose-dependent fashion with IC_50 _values ranging from 15 μM to 51 μM. The ability of α-TEA to kill breast cancer cells irrespective of HER2/*neu *status suggests that it could be effective for treating HER2/*neu*^+ ^as well as HER2/*neu*^- ^breast cancer.

### Enhanced tumor cell killing by α-TEA + anti-HER2/*neu *antibody treatment

**Figure 2:**
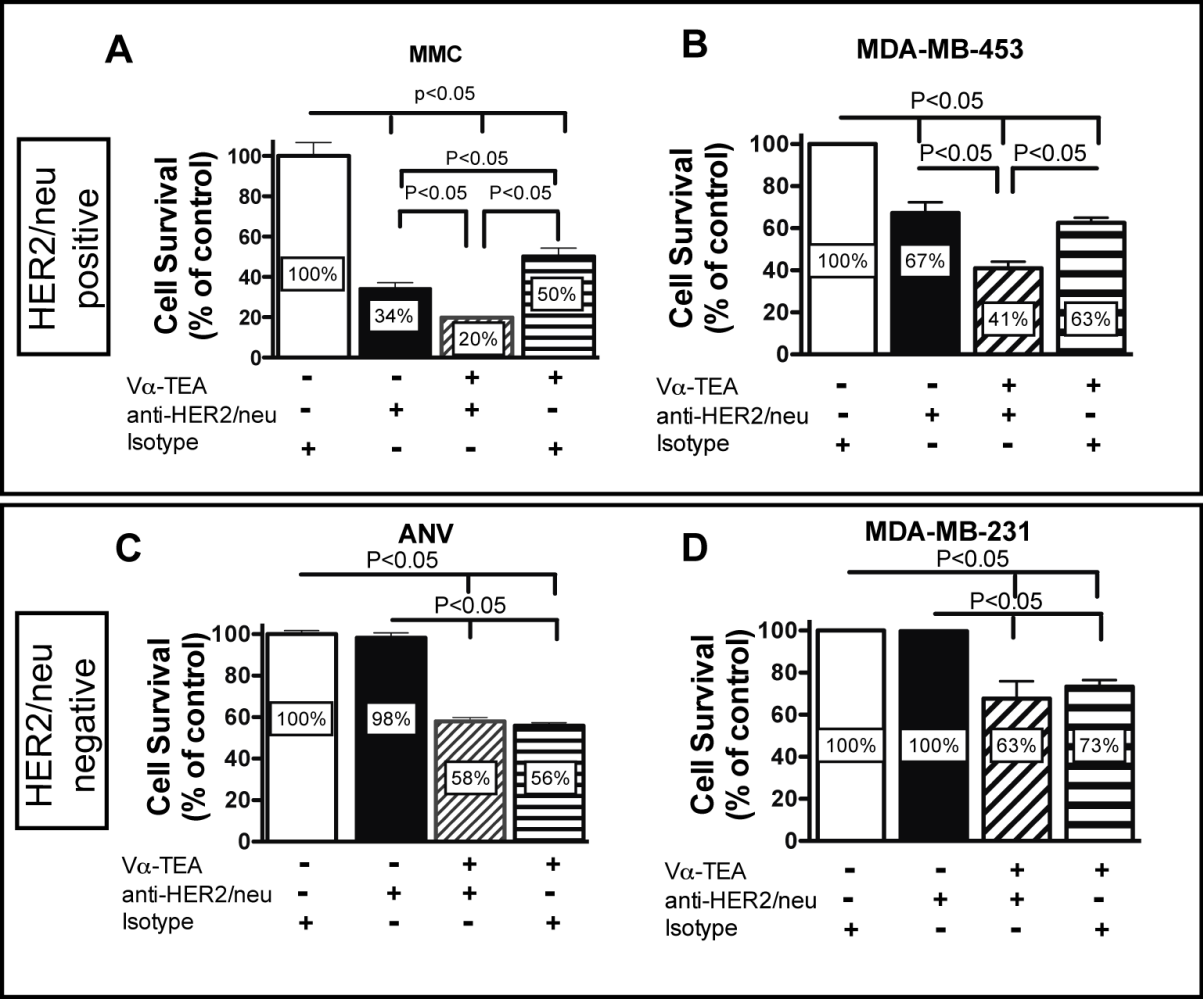
**Susceptibility of HER2/*neu *positive and negative tumor cell lines to α-TEA + anti-HER2/*neu *antibody**. MMC (HER2/*neu*^+^) or ANV (Her2/*neu*^-^) cells were allowed to adhere overnight. MMC and ANV cells were then treated with 20 μg/mL of anti-HER2/*neu *antibody (7.16.4), 20 μg/mL of mouse IgG2a antibody (isotype) or 20 μM of Vα-TEA. MDA-MB-453 (HER2/*neu*^+^) and MDA-MB-231 cells were treated with 10 μg/mL (MDA-MB-453) or 20 μg/mL (MDA-MB-231) trastuzumab or rituximab (isotype) or 10 μM Vα-TEA. After a 5 day exposure, cell survival was determined by MTT assay. Combined results from two to three independent experiments are expressed as mean percentage ± SD of untreated cells (isotype) cells.

We next compared tumor cytotoxicity induced by α-TEA + anti-HER2/*neu *antibody using both murine HER2/*neu*^+ ^(MMC) and HER2/*neu*^- ^(ANV) and human HER2/*neu*^+ ^(MDA-MB-453) and HER2/*neu*^- ^(MDA-MB-231) breast cancer cell lines. To achieve this goal, tumor cells were treated with sub-optimal doses of α-TEA alone, anti-HER2/*neu *antibody alone or α-TEA + anti-HER2/*neu *antibody. The data show that combining a sub-optimal dose of α-TEA with HER2/*neu*-specific antibody resulted in an enhanced cytotoxic effect on the HER2/*neu*^+ ^breast cancer cell lines (Figure 2A and 2B). This is in contrast to the HER2/*neu*^- ^cell lines where we observed no significant reduction in tumor cell survival in anti-HER2/*neu *antibody-treated cells and no enhancement of tumor cell growth reduction beyond the α-TEA-mediated effect (Figure 2C and 2D).

### α-TEA plus trastuzumab combination treatment induces apoptosis and reduces pro-survival signaling

**Figure 3:**
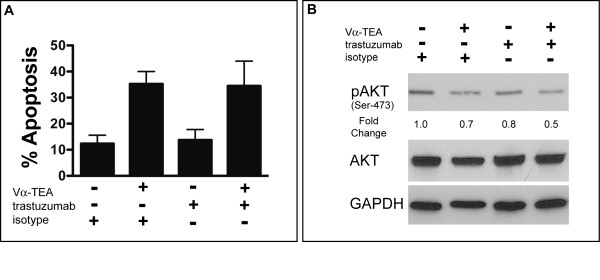
**Mechanism of α-TEA and trastuzumab-mediated tumor cell inhibition**. (A) MDA-MB-453 tumor cells were treated with Vα-TEA and trastuzumab (or isotype antibody) for 72 hours. Cells were collected and analyzed for apoptosis by PE-Annexin-V stain. Mean frequency ± SD of apoptotic cells (PE-Annexin-V positive) is shown from three independent experiments. (B) MDA-MB-453 tumor cells were treated with Vα-TEA and trastuzumab (or isotype antibody) for 24 hours. Phosphorylated-(Ser473)-AKT, total AKT and GAPDH levels were determined by western immunoblotting. Numbers indicate fold-change over untreated (isotype-treated) cells.

Previously it has been shown by us [6,7] and others [3,4] that α-TEA-mediated tumor cell cytotoxicity is partially mediated through the induction of apoptosis. In order to determine if α-TEA also induced apoptosis in the human HER2/*neu*-expressing MDA-MB-453 tumor cell line, we examined Annexin-V staining after α-TEA treatment. The data in Figure 3A show that α-TEA treatment increased the fraction of apoptotic cells approximately 3-fold compared to untreated (isotype-treated) cells. Trastuzumab alone did not induce apoptosis of MDA-MB-453 cells. Furthermore, there was no increase in apoptotic cells when cells were treated with α-TEA + trastuzumab compared to α-TEA treatment. One of the mechanisms of trastuzumab-mediated tumor cell reduction is the inhibition of pro-survival signaling through the phosphatidylinositol 3-kinase (PI3K) pathway. HER2/*neu *over expression leads to activation of PI3K that activates AKT by phosphorylation. AKT exerts its survival role via a diverse array of substrates, which control key cellular processes, including apoptosis, cell cycle progression, transcription, and translation [36]. Therefore AKT phosphorylation status (pAKT) was used as a surrogate marker to determine the effect of trastuzumab on MDA-MB-453 tumor cells. The data (Figure 3B) show that trastuzumab treatment resulted in decreased pAKT levels, suggesting reduced signaling through HER2/*neu*. Previously, it has been shown that α-TEA reduced tumor cell viability not only by activation of pro-apoptotic pathways but also by reducing PI3K-mediated growth/survival signaling [8,37]. α-TEA treatment also decreased pAKT levels compared to untreated cells (Figure 3B). More importantly, the data show that the combination of α-TEA+trastuzumab reduced pAKT level the most, resulting in a 50% loss of activated AKT compared to untreated cells (Figure 3B). Taken together, these results suggest that, while the major mechanism of α-TEA+trastuzumab-mediated tumor cell reduction *in vitro *is the induction of apoptosis by α-TEA, inhibition of pro-survival signaling through AKT may also contribute to the superior cytotoxic effect of the combination treatment.

### α-TEA plus trastuzumab combination therapy suppresses human breast cancer and leads to complete tumor regression

**Figure 4:**
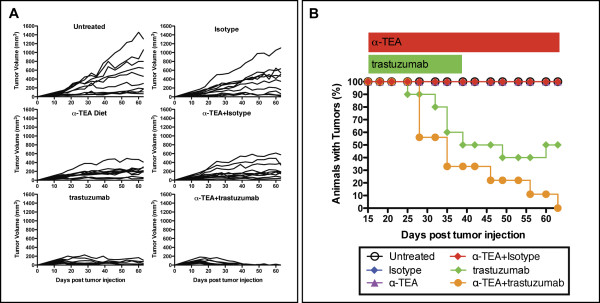
**Tumor regression by α-TEA plus trastuzumab treatment**. SCID mice (n = 9 to 10 mice per group) received a s.c. injection of MDA-MB-453 cells. After tumor establishment (day 15 post-tumor injection), mice received α-TEA in the diet (~2 mg/day/mouse) until day 63 (red bar) or 40 μg trastuzumab by i.p. injection, every 2 to 3 days for a total of 12 injections until day 40 (green bar). One mouse in the α-TEA + trastuzumab group died of unknown cause on day 35 post-tumor cell injection and was excluded from analysis. (A) Individual tumor volumes. (B) Tumor frequency.

We next wanted to test the efficacy of concurrent α-TEA and trastuzumab (Herceptin^®^) treatment in suppressing established HER2/*neu*^+ ^human mammary cancer. To achieve this goal, SCID mice were injected with 1 × 10^7 ^HER2/*neu*-expressing MDA-MB-453 tumor cells. Following tumor establishment on day 15, mice were randomized and placed on α-TEA-supplemented mouse chow and trastuzumab therapy was initiated. The mice received 12 i.p. injections of trastuzumab (40 μg/injection) given 3 times a week until day 40 post-tumor injection. This dose of trastuzumab (2 mg/kg) has been previously reported to inhibit tumor growth in xenograft models [38] and is equivalent to the maintenance dose (2 mg/kg) administered to breast cancer patients [39]. The data (Figure 4A) show that when compared to mice on the control diet (untreated or isotype-treated), α-TEA (+/- isotype) or trastuzumab individually inhibited growth of established MDA-MB-453 tumors. By day 40, tumors had completely regressed in 5 of 10 mice (50%) in the trastuzumab group compared to 0 of 10 in the α-TEA group. The anti-tumor response was most robust in the α-TEA + trastuzumab group where tumor regression occurred in 6 of 9 animals (67%) by day 40. At the end of α-TEA treatment (day 63 post-tumor injection), the tumor sizes of the remaining mice in the trastuzumab group (50%) were approximately maintained at pre-treatment levels (Figure 4B). This is in contrast to the combination treatment (α-TEA + trastuzumab) that continued to cause tumor regression resulting in 9 of 9 tumor-free mice (100%) by the end of α-TEA treatment (day 63). Tumor recurred in one mouse in this group 7 days after discontinuation of α-TEA diet (data not shown). The percentage of tumor-free animals in the α-TEA + trastuzumab group was significantly higher than in the trastuzumab alone group (p = 0.033) and all other treatment groups (p < 0.0001 for all comparisons). In addition, the rate at which the percentage of animals with tumors decreased from day 15 to 63 was significantly greater for the α-TEA + trastuzumab group (p = 0.029) compared to the trastuzumab alone group (Figure 4B). Accordingly, the rate at which the tumor volume decreased from day 15 to 63 was also significantly greater for the α-TEA + trastuzumab group (p = 0.030) compared to the trastuzumab group.

### Effect of α-TEA plus trastuzumab treatment on HER2/*neu *expression *in vivo*

**Figure 5:**
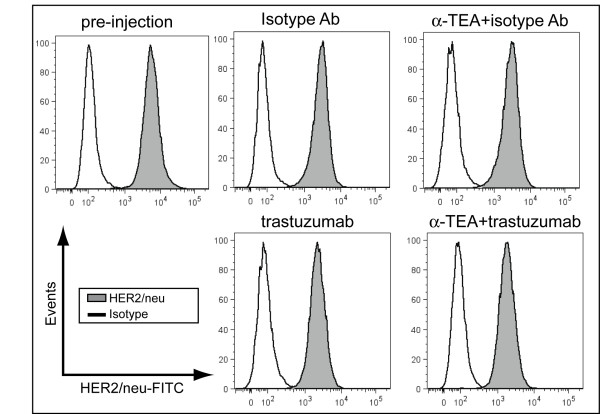
**α-TEA inhibits tumor growth without modulating HER2/*neu *expression**. SCID mice with established MDA-MB-453 tumors (day 15 post-tumor injection) received α-TEA in the diet (~2 mg/day/mouse) or 40 μg trastuzumab by i.p. injection, every 2 to 3 days for a total of 7 injections. On day 30 post-tumor injection, tumors were mechanically dissociated and a single cell suspension was prepared and analyzed for HER2/*neu *expression by flow cytometry. HER2/*neu *expression of MDA-MB-453 tumor cells used for transplantation is shown for comparison (pre-injection). Cells were gated on light scatter and non-viable cells were excluded from the analysis using viability stain.

The efficacy of trastuzumab depends on the stability of HER2/*neu *expression on breast cancer during intervening trastuzumab or adjuvant chemotherapy treatment. To address the concern that trastuzumab, or the α-TEA+trastuzumab combination treatment may result in down regulation of HER2/*neu*, we determined HER2/*neu *expression on tumor cells recovered from mice after 15 days of α-TEA+trastuzumab therapy (day 30 post-tumor injection). Cell surface HER2/*neu *expression remained high on tumor cells after trastuzumab, α-TEA or α-TEA+trastuzumab treatment (Figure 5) compared to tumor cells from isotype-treated mice and HER2/*neu *expression levels of tumor cells at the time of tumor implantation (pre-injection).

### Effect of α-TEA plus trastuzumab on *in vivo *tumor proliferation and apoptosis induction

**Figure 6:**
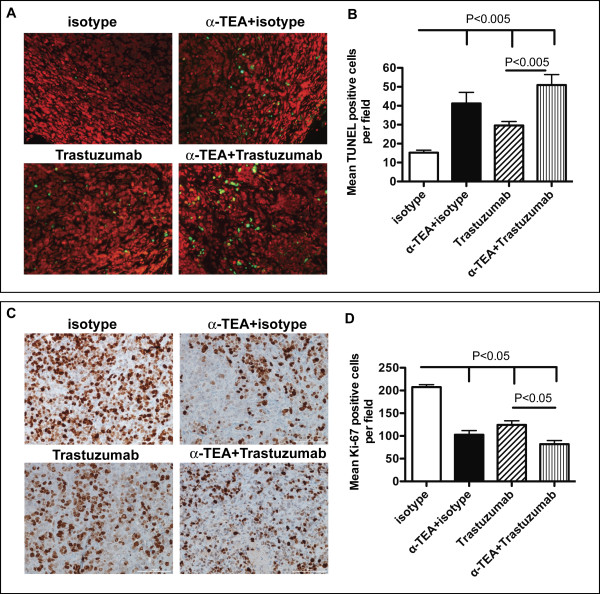
**Immunohistologic analysis of tumor tissue after α-TEA plus trastuzumab treatment**. SCID mice with established MDA-MB-453 tumors (day 15 post-tumor injection), mice received α-TEA in the diet (~2 mg/day/mouse) or 40 μg trastuzumab by i.p. injection, every 2 to 3 days for a total of 7 injections. On day 30 post-tumor injection tumors were resected. (A) Tumors were examined for apoptosis by TUNEL assay. Representative images of tumor sections (20 × magnification) showing FITC-TUNEL-positive (green) and propipdium iodide counterstained (red) cells. (B) Mean number of positive stained cells per field (40 × magnification) in sections from 3 mice per group. (C) Tumors were examined for proliferation by Ki-67 immunostain. Representative images of tumor sections (20 × magnification) showing Ki-67-positive (brown) and hematoxylin counterstained (blue) cells. (D) Mean number of positive stained cells per field (40 × magnification) in sections from 3 mice per group.

In order to determine the mechanism of α-TEA+trastuzumab-mediated tumor suppression *in vivo*, we assessed *in situ *apoptosis and proliferation in tumors of three randomly selected mice per treatment group. Because α-TEA+trastuzumab therapy resulted in a 100% cure rate on day 63 post-tumor injection, tumors were obtained from mice at an intermediate time point (day 30 post-tumor injection) after 15 days of α-TEA+trastuzumab treatment. Serial sections of each tumor in the isotype-treated, α-TEA, trastuzumab and α-TEA+trastuzumab treatment groups were histochemically stained with hematoxylin & eosin (H&E), analyzed for apoptosis by TUNEL assay (Figure 6A and 6B) or immunostained with Ki-67-specific antibody (Figure 6C and 6D).

All tumors had a morphologic diagnosis of carcinoma, and displayed a similar pattern with multiple lobules composed of solid sheets of cells separated by variably dense fibrovascular stroma. Bi- and occasionally multi-nucleate cells and numerous atypical mitotic figures were present. Tumors varied in the amount of average number of mitoses per high powered field (mitotic index). Tumors in the isotype-treated group had mitotic indices in excess of 10. Tumors in the trastuzumab group had mitotic indices of 7 or more and tumors from the α-TEA-treated group had mitotic indices between 5 and 10. In contrast, Tumors from the α-TEA+trastuzumab group had the lowest mitotic indices of 4 and 5. Analysis of *in situ *apoptosis showed that compared to isotype-treated mice, α-TEA, trastuzumab and α-TEA+trastuzumab treatment resulted in significant increases of TUNEL positive cells per field (Figure 6B). Compared to isotype-treated mice, α-TEA alone resulted in 2.7-fold increase of TUNEL positive cells per field, while trastuzumab alone resulted in a more moderate 2-fold increase of TUNEL positive cells (Figure 6B). The combination treatment resulted in the highest amount of apoptosis resulting in a 3.3-fold increase over isotype-treated mice. Immunostains with Ki-67-specific antibody (Figure 6D) revealed significant reductions of Ki-67 positive cells in all treatment groups compared to the isotype-treated group ranging from 1.7-fold (trastuzumab) to 2.5-fold (α-TEA+trastuzumab) reduction. Taken together, these data suggest that the mechanism of tumor regression is a combination of induction of apoptosis and reduced tumor cell proliferation.

## Discussion

Despite recent advances in treatment, HER2/*neu *positive or negative breast cancer continues to be a major cause of death in women. α-TEA is an orally active semi-synthetic analog of vitamin E, which has demonstrated anti-tumor activity against several breast cancers *in vitro *and *in vivo*. Trastuzumab (Herceptin^®^), a humanized antibody that targets the extracellular domain of HER2/*neu*, has become standard of care for the treatment of HER2/*neu *over-expressing early stage and metastatic breast cancer. However, trastuzumab is effective in only a small percentage of patients with high HER2/*neu*^+ ^tumors resulting in only a 15-30% response rate [18,21], which can be significantly increased to 50-80% by the addition of chemotherapeutic drugs [40]. In this study we evaluated a novel combination therapy modality consisting of oral α-TEA and HER2/*neu*-specific antibody. Our data demonstrate the capability of α-TEA to efficiently kill mouse mammary and human breast cancer cell lines irrespective of the expression level of HER2/*neu*. This finding suggests that HER2/*neu *expression is not required for α-TEA-mediated tumor cytotoxicity and that HER2/*neu*-independent pathways may be operative in α-TEA-mediated killing of HER2/*neu*-expressing tumor cells. Furthermore, the ability of α-TEA to kill breast cancer cells irrespective of HER2/*neu *status suggests that it could be effective for treating HER2/*neu*^+ ^as well as HER2/*neu*^- ^breast cancers. This finding led us to hypothesize that combining α-TEA with trastuzumab will result in an enhanced antitumor response. Our data demonstrate a direct correlation between HER2/*neu *expression and susceptibility to HER2/*neu*-specific antibody. The MMC and MDA-MB-453 cell lines exhibiting high HER2/*neu *expression were susceptible to HER2/*neu *antibody treatment. Combining sub-optimal doses of α-TEA (20 μM for MMC, and 10 μM for MDA-MB-453) with HER2/*neu*-specific antibody resulted in an enhanced cytotoxic effect against the HER2/*neu*^+ ^cell lines that was absent in the HER2/*neu*^- ^cell lines (ANV and MDA-MB-231). This result suggests that lower doses of α-TEA may be therapeutic if combined with HER2/*neu*-specific antibody. The modest efficacy of the combination treatment *in vitro *is not surprising since the major *in vitro *mechanism of trastuzumab is thought to be growth inhibition, while the major *in vivo *mechanism of trastuzumab anti-tumor activity, antibody-dependent cell-mediated cytotoxicity [22,41] and cross-priming by antigen-presenting cells (APC), are lacking in the *in vitro *system. This is corroborated by our results that trastuzumab *in vitro *did not induce apoptosis in MDA-MB-453 tumor cells but reduced the levels of activated AKT which is an important survival and growth signal, suggesting that the main trastuzumab effect *in vitro *is anti-proliferative. In contrast, α-TEA treatment *in vitro *resulted in significant induction of apoptosis but also cooperated with trastuzumab to further decrease activated AKT levels, providing a mechanism of the enhanced tumor cell reduction in the α-TEA+trastuzumab combination treatment. Our finding of increased apoptosis and decreased proliferation in tumor tissue after α-TEA and/or trastuzumab treatment is also consistent with the notion that the major mechanism of anti-tumor efficacy of the combination treatment *in vivo *is the result of the accumulation of apoptotic cell death and restricted proliferation.

Using a xenograft model of human breast cancer, we demonstrated for the first time that the combination of α-TEA plus trastuzumab led to complete tumor regression in all treated animals compared to 50% regression in mice that received trastuzumab alone. The enhancement of the *in vivo *anti-tumor response in the combination group reinforces the *in vitro *studies using HER2/*neu*^+ ^mouse mammary and human breast cancer cell lines. An important aspect of the combination therapy is that anti-tumor activity was detected using a trastuzumab dose that is within the dose range administered to HER2/*neu*^+ ^breast cancer patients [39], highlighting the translational potential of this novel combination therapy. Evaluation of HER2/*neu *expression at an intermediate time point during the treatment regimen revealed no modulation of HER2/*neu *expression. This finding suggests that ongoing α-TEA or α-TEA+trastuzumab treatment of established tumors does not impair HER2/*neu *expression, allowing for continued concurrent treatment of such tumors with HER2/*neu*-specific antibody.

The anti-tumor effect in the α-TEA, trastuzumab and the combination groups correlated with increased levels of *in situ *apoptosis and decreased proliferation in the tumors compared to the c group. The combination of α-TEA+trastuzumab resulted in the highest level of *in situ *apoptosis and the lowest level of proliferating cells that correlated with the faster rate of tumor regression in the combination group compared to the trastuzumab alone group. These data suggest that induction of apoptosis and reduction of tumor cell proliferation is a major mechanism of breast cancer suppression by α-TEA+trastuzumab therapy *in vivo*.

## Conclusions

In summary, these findings demonstrate for the first time, the efficacy of a stable orally active vitamin E derivative (α-TEA) plus HER2/*neu*-specific antibody in treating established HER2/*neu *positive breast cancer and highlight the potential usefulness of α-TEA, a relatively non-toxic chemotherapeutic agent, for treating HER2/*neu *positive or negative breast cancer.

## List of abbreviations

ANOVA: one-way analysis of variances; α-TEA: alpha-tocopheryloxyacetic acid; α-TOH: alpha-tocopherol; α-TOS: alpha-tocopheryl succinate; DMEM: Dulbecco's Modified Eagle medium; DMSO: Dimethyl sulfoxide; FBS: fetal bovine serum; HBSS: Hank's balanced salt solution; MTT: Thiazyl Blue Tetrazolium Bromide; NK: natural killer cell; RPMI-1640: Roswell Park Memorial Institute-1640 medium; TUNEL: terminal deoxynucleotidyl transferase-mediated dUTP nick end labeling; VEA: vitamin E analog.

## Competing interests

The authors declare that they have no competing interests.

## Authors' contributions

TH participated in the design of the study, performed *in vitro *and *in vivo *experiments including characterization of HER2/*neu *expression levels, α-TEA susceptibility and xenograft experiments. DJB-D conducted some α-TEA susceptibility experiments and coordinated the *in vivo *xenograft experiment. BBC conducted some α-TEA susceptibility experiments. TM conducted *in vitro *apoptosis analysis. DGB conducted the histopathologic analysis of tumor sections. LHH, Dv-H and SG provided intellectual and material support. MLD, HL, and MLP provided material support and participated in critical discussions. EW participated in critical discussions. ETA conceived of the study, and participated in its design and draft of the manuscript. All authors read and approved the final manuscript.

## Pre-publication history

The pre-publication history for this paper can be accessed here:

http://www.biomedcentral.com/1471-2407/11/471/prepub
